# Garcinone E Mitigates Oxidative Inflammatory Response and Protects against Experimental Autoimmune Hepatitis via Modulation of Nrf2/HO-1, NF-κB and TNF-α/JNK Axis

**DOI:** 10.3390/nu15010016

**Published:** 2022-12-21

**Authors:** Gamal A. Mohamed, Sabrin R. M. Ibrahim, Rawan H. Hareeri, Lenah S. Binmahfouz, Amina M. Bagher, Hossam M. Abdallah, Wael M. Elsaed, Dina S. El-Agamy

**Affiliations:** 1 Department of Natural Products and Alternative Medicine, Faculty of Pharmacy, King Abdulaziz University, Jeddah 21589, Saudi Arabia; 2 Department of Chemistry, Preparatory Year Program, Batterjee Medical College, Jeddah 21442, Saudi Arabia; 3 Department of Pharmacognosy, Faculty of Pharmacy, Assiut University, Assiut 71526, Egypt; 4 Department of Pharmacology and Toxicology, Faculty of Pharmacy, King Abdulaziz University, Jeddah 21589, Saudi Arabia; 5 Department of Anatomy and Embryology, Faculty of Medicine, Mansoura University, Mansoura 35516, Egypt; 6 Department of Pharmacology and Toxicology, Faculty of Pharmacy, Mansoura University, Mansoura 35516, Egypt

**Keywords:** *Garcinia mangostana*, Clusiaceae, xanthones, garcinone E, hepatoprotective, concanavalin A, hepatitis, industrial development

## Abstract

*Garcinia mangostana* L. (Clusiaceae), a popular tropical fruit for its juiciness and sweetness, is an opulent fountain of prenylated and oxygenated xanthones with a vast array of bio-activities. Garcinone E (GE), a xanthone derivative reported from *G. mangostana,* possesses cytotoxic and aromatase inhibitory activities. The present research endeavors to investigate the hepato-protection efficaciousness of GE on concanavalin-A (Con-A)-instigated hepatitis. Results showed that GE pretreating noticeably diminishes both the serum indices (transaminases, ALP, LDH, and γ-GT) and histopathological lesions of the liver. It counteracted neutrophil and CD4+ infiltration into the liver. GE furthered the Nrf2 genetic expression and its antioxidants’ cascade, which resulted in amelioration of Con-A-caused oxidative stress (OS), lipid per-oxidative markers (4-HNE, MDA, PC) reduction, and intensified antioxidants (TAC, SOD, GSH) in the hepatic tissue. Additionally, GE prohibited NF-ĸB (nuclear factor kappa-B) activation and lessened the genetics and levels of downstream cytokines (IL1β and IL6). Moreover, the TNF-α/JNK axis was repressed in GE-treated mice, which was accompanied by attenuation of Con-A-induced apoptosis. These findings demonstrated the protective potential of GE in Con-A-induced hepatitis which may be associated with Nrf2/HO-1 signaling activation and OS suppression, as well as modulation of the NF-κB and TNF-α/JNK/apoptosis signaling pathway. These results suggest the potential use of GE as a novel hepato-protective agent against autoimmune hepatitis.

## 1. Introduction

Auto-immune hepatitis (AIH) is a grave chronic hepatic inflammation disorder, which is related to an immune imbalance of T cells and macrophages [1]. Its manifestations include ample hepatic inflammation, necrosis, and apoptosis that may give rise to hepatic failure in nearly 1% of AIH patients. Even now, glucocorticoids and immunosuppressants are the standard conventional therapy for AIH despite their deleterious side effects. Hence, it is mandatory to explore safer and more specific therapeutic candidates [2]. Con-A (concanavalin-A; a glucose/mannose-binding plant lectin) is a widely employed model to induce an AIH in mice that closely mimics the features of human AIH. Con-A injection provokes CD4 T cell activation, differentiating and ensuing the release of various inflammation mediators (i.e., TNF-α (tumor-necrosis-factor-alpha), ILs (interleukins)) [3,4]. Many pathogenic inflammatory and apoptotic signaling pathways interact and control the Con-A-produced AIH pathogenesis. Previous investigations have documented the vital function of NF-ĸB (nuclear factor-κB) in mediating Con-A inflammatory hepato-toxic reactions. It was reported that Con-A induces the activation of NF-ĸB and its nuclear translocation where it enhances the expression and release of downstream cytokines as TNF-α [5,6,7]. Controlling the inflammatory NF-ĸB pathway is a potential target for new anti-inflammatory therapeutics [8]. Besides the inflammation, apoptosis is critically involved in Con-A-caused hepatitis pathogenesis. Following Con-A injection, hepatocytes experience apoptosis owing to pro-apoptotic protein (Bax and Bad) upregulation and anti-apoptotic (Bcl2 and Bcl-xl) protein downregulation [9].

Recently, JNK (a member of the mitogen-activated protein kinases (MAPKs) family) has grabbed the attention of many researchers for its important role in the pathogenic events of Con-A-induced hepatitis. Con A injection potentiates JNK phosphorylation and its translocation into the mitochondrial membrane or cell nucleus, resulting in hepatic damage [10,11]. Moreover, TNF-α/JNK signaling is linked to the activation of such pro-apoptotic genes as Bax and the initiation of caspase-dependent apoptosis [12]. The interplay between inflammatory and apoptotic signaling, along with the depression of antioxidative protective pathways such as Nrf2 (nuclear factor erythroid 2-related factor 2) exacerbates Con-A-boosted hepatic oxidative injury [13]. Thus, agents that affect the crosstalk between these signaling pathways can confer protection against Con-A-induced AIH.

Herbal medicines have become a promising therapy for hundreds of years for treating various liver disorders [14]. Many of the therapeutically used agents in liver diseases are either natural products or their derivatives because of their capacity to act on various biological targets [15]. Moreover, many natural compounds, extracts, and herbal formulations have been proven to ameliorate Con-A-induced hepatitis [16]. *Garcinia mangostana* L. (GM, mangosteen, Queen of fruits, Clusiaceae), amongst the most common tropical fruits, is popular for its juiciness and sweetness, plus its importance in promoting people’s health [17,18,19,20,21,22]. It is an opulent fountain of xanthones [23,24,25,26]. In Southeast Asian traditional folk medicine, GM is utilized for treating typhoid, diarrhea, ulcers, sprains, abdominal pain, leukorrhea, gonorrhea, inflammation, and skin infections, as well as for its therapeutic effects, such as antimicrobial and antiparasitic properties, and wound healing [27,28]. In the U.S. market, it is a popular antioxidant dietary supplement, and its juice was ranked as one of the top three-selling “botanicals single” [29]. *G. mangostana*’s xanthones have demonstrated interesting bioactivities, such as anti-proliferative, antioxidant, anti-inflammatory, anti-nociceptive, pro-apoptotic, anti-obesity, antiplasmodial, anti-microbial, and anti-carcinogenic activities [17,18,19,20,21,22,30,31,32]. In our continuing interest to estimate the bioactivities of GM xanthones, GE was purified and appraised for its hepato-protection effectiveness on Con-A-produced AIH. Moreover, the possible pathways involved in the GE-protective effects were evaluated.

## 2. Materials and Methods

### 2.1. General Experimental Procedures

A spectrometer JEOL JMS-SX-SX-102A was employed for EIMS. INOVA-850 BRUKER Unity was utilized for recording NMR. TLC plates SiO_2_60-F_254_ and SiO_2_60/RP-18 were utilized for TLC and chromatographic analyses, respectively. 

### 2.2. Plant Material

In December 2019, *G*. *mangostana* was procured from a Saudi local market. Its authentication was accomplished as previously stated [18], and a voucher specimen (no. GM1724) was kept in the herbarium at the Faculty of Pharmacy, KAU.

### 2.3. Extraction and Isolation

An acetone extract (GMA, 74 g) was prepared by extracting the pericarps (1.5 kg) with acetone (4 L × 5), then evaporation [15,18,33]. GMA extract SiO_2_ CC utilizing EtOAC/*n*-hexane afforded GMA-1:GMA-9 subfractions. Using an authentic sample of garcinone E, subfractions GMA-3 and GMA-4 (11.4 g) were chromatographed on a SiO_2_ column (300 × 100 × 3 cm) using *n*-hexane/EtOAc (95/5-70/30) to get garcinone E that was purified on RP-18 CC (MeOH/H_2_O gradient) to yield garcinone E (435 mg).

### 2.4. Animals 

Male BALB/c mice (20–22 g) were retained under standard conditions of humidity, temperature, and light/dark cycle. Mice were permitted to freely access standard laboratory rodent water and food. Approval of the study protocol was given by the Research Ethics Committee (PH-115-40, Faculty of Pharmacy, KAU) and the procedures were carried by exacting standards of Saudi Arabia for the Care and Use of Laboratory Animals.

### 2.5. Chemicals and Reagents

Con-A was dissolved in phosphate-buffered saline (PBS 1×) (Sigma–Aldrich/St. Louis/MO/USA). AST, ALT, LDH, and ALP colorimetric kits were provided by Human (Wiesbaden/Germany), while γ-GT was from Spectrum Co. (Cairo, Egypt). A Caspase-3 assay kit was secured by Sigma–Aldrich (USA). MDA, NO, SOD, GSH, and TAC kits were supplied by Bio-Diagnostic Co. (Giza, Egypt). ELISA kits for myeloperoxidase (Thermo Fisher Scientific/Waltham/MA/USA), 4-HNE (4-hydroxynonenal), PC (protein carbonyl), HO-1 (heme-oxygenase-1), Bax (Bcl2 Associated X Protein), Bcl2, C-Jun N-Terminal Kinase (JNK) (MyBioSource Inc./San Diego/CA/USA), NF-ĸB p65 (Abcam/UK), TNF-α and IL-6 (R&D System/Minneapolis/MN/USA) were purchased. A TransAM Nrf2 kit (Active Motif Inc./Carlsbad/CA/USA), RNeasy MiniKit and cDNA-reverse transcription kit were also purchased (Qiagen/Germantown/MD/USA).

### 2.6. Experimental Design

Mice were assigned into 5 groups (8 mice each) in a random way: 

Control group: Received intravenous PBS;

GE group: Administered GE (50 mg/kg/once daily/5 days);

Con-A group: Received Con-A (15 mg/kg, IV) [9,34]; 

GE 25 mg/kg + Con-A: Received GE (25 mg/kg/5 sequential days) prior to Con-A injection;

GE 50 mg/kg + Con-A: Received GE (50 mg/kg/5 sequential days) prior to Con-A injection. 

Twelve hours post-Con-A injection, mice were humanely killed under anesthesia. Liver and blood samples were gathered. After centrifuging the blood, the obtained serum samples were preserved for further investigation at −80 °C. Homogenization of liver tissue (from left median lobe) and then centrifugation for 15 min at 4 °C were done to get the supernatants which were kept at −80 °C for further analysis.

### 2.7. Biochemical Measurements

#### 2.7.1. Hepatotoxicity Markers

Serum AST, ALT, LDH, ALP, and γ-GT were estimated under the manufacturers’ instruction manuals.

#### 2.7.2. MPO

This was assessed according to the manufacturer’s instruction kit in liver homogenate.

#### 2.7.3. Antioxidant Balance and Oxidative Stress 

MDA, 4-HNE, PC, SOD, GSH, and TAC levels were determined in different groups’ hepatic supernatants relying on the kits’ instructions.

#### 2.7.4. Nitric Oxide (NO)

This was determined following the procedures of the kit. Briefly, the hepatic tissue was homogenized using cold phosphate buffer containing EDTA (2 mM) and then centrifuged (4000 rpm) for 15 min at 4 °C to collect the supernatants. In an acidic medium, the supernatants were mixed with sulphanilamide and N-(1–naphthyl) ethylenediamine. The reddish-purple product was quantified spectrophotometrically at 540 nm.

### 2.8. ELISA Assay

#### 2.8.1. Nrf2 and HO-1

A nuclear extract was obtained from hepatic tissue to estimate Nrf2 binding activity as described in the kit’s protocol. In brief, a small piece of fresh hepatic tissue was mixed with hypotonic ice-cold extraction buffer having detergent, dithiothreitol, and protease and phosphatase inhibitors. After 15 min of incubation, the mixture was centrifuged for 10 min at 4 °C. Obtained cell pellets were resuspended and incubated for 15 min on ice with a vortex every 3 min. Another centrifugation at 4 °C was done to obtain the supernatants and their protein content was determined. HO-1 was measured in hepatic supernatants according to the kit’s instructions.

#### 2.8.2. Inflammatory and Apoptosis Mediators

Levels of NF-κB, IL-6, IL-1β, TNF-α, JNK, BcL2, caspase-3, and Bax were determined in the hepatic supernatants in line with the instruction of the kits. 

### 2.9. Histopathology 

A liver piece was fixed and then embedded in paraffin. Specimen (4–5 μm) staining was performed with H&E (hematoxylin and eosin; MuseChem, Fairfield, NJ, USA) as previously described by Feldman and Wolfe [35]. In terms of inflammation and necrosis, hepatic lesions were graded (4: severe (≥60%); 3: moderate (≤60%); 2: mild (≤30%); 1: very mild; 0: none) [5].

### 2.10. IHC (Immunohistochemistry) 

Automatic IHC-staining was carried out utilizing the BenchMark Ventana XT system (Ventana Medical Systems/Tucson/AZ). In brief, after dewaxing of tissue samples and rehydration, samples’ peroxidases were neutralized using hydrogen peroxide (3%). The liver sections were immuno-stained utilizing primary antibodies: rabbit polyclonal antibody to CD4 (1:100, Abnova/Taipie/Taiwan), PCNA (1:100, Abcam/Cambridge/UK), and NF-κBp65 (1:200, Fisher Scientific Inc./Waltham/MA/USA), as formerly described [36]. Slides were visualized using diaminobenzidine and counterstained. The analysis of immuno-stain percentage was done using ImageJ, NIH.

### 2.11. RT-PCR 

Initially, total RNA was separated from the hepatic tissue (≈20 mg) utilizing a RNeasy MiniKit according to its guidelines. The RNA samples’ purity was checked utilizing a A260/A280 ratio, and only pure samples (ratio of 1.8–2.1) were converted to cDNA using a cDNA-reverse transcription kit. RT-PCR was done utilizing PCR Master Mix SYBR Green for HO-1, Nrf2, GCLc, IL-6, NF-κB, IL-1β, and TNF-α. In Table 1, the listed primers are shown, which were designated using Primer3Plus software and a Primer-BLAST program. mRNA expression quantification was normalized against β-actin utilizing the double-delta Ct method.

### 2.12. Statistical Analysis

The findings are shown as mean values and standard error (SE) of the mean. One-way ANOVA and post hoc Tukey–Kramer multiple comparing tests were utilized for comparing different groups. *p* < 0.05 was assigned as being statistically significant.

## 3. Results

**Figure 1:**
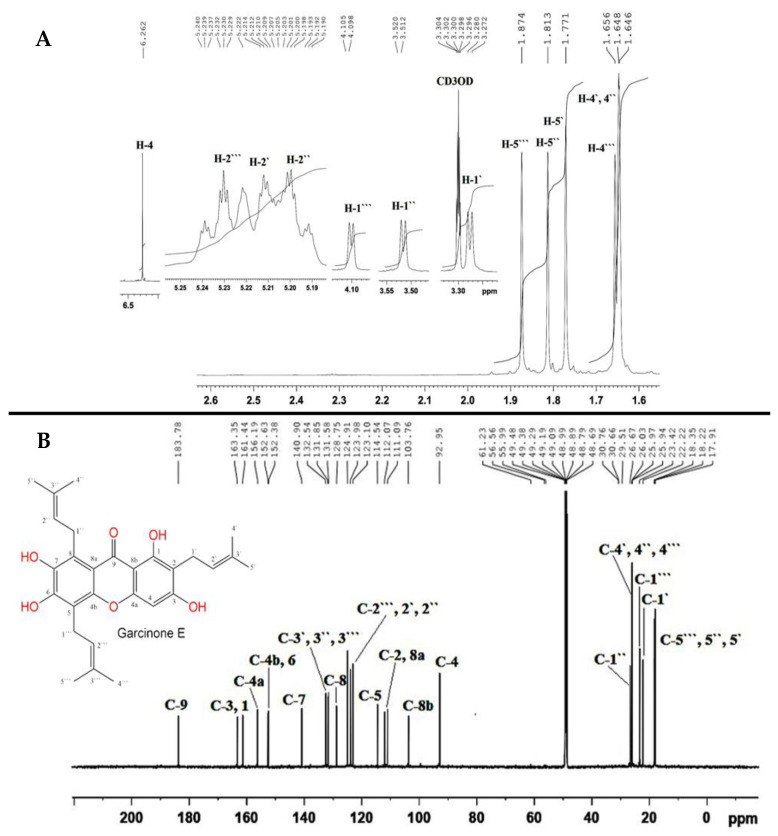
^1^H (**A**) and ^13^C NMR (**B**) spectra of GE (850 and 214 MHz, CD_3_OD).

The EtOAc extract of *G. mangostana* was subjected to various chromatographic techniques to afford GE as a yellow powder which was identified based on its physical and spectral data and in comparison with the literature [37] (Figure 1A,B).

The GE-treated group exhibited no significant change compared to normal control mice regarding all measurements.

### 3.1. GE-Alleviated Con-A-Induced Hepatic Injury

**Figure 2:**
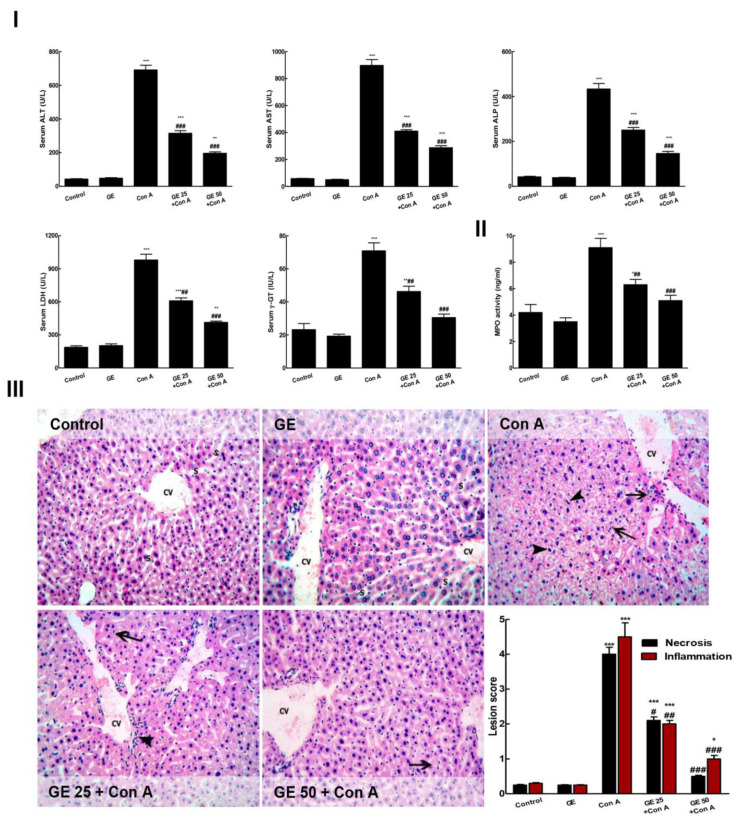
Garcinone E (GE) alleviated concanavalin A (Con A)-induced hepatic injury. (**I**). ALT (Serum alanine amino-transferase), AST (aspartate amino-transferase), ALP (alkaline phosphatase), γ-GT (gamma-glutamyl-transferase), and LDH (lactate dehydrogenase). (**II**). Hepatic MPO (myeloperoxidase). (**III**). Hepatic sections stained with H&E (×200); Control and GE groups showed normal liver architecture with the hepatocytes arranged in regular plates separated by the blood sinusoids (S) and radiating from around the CV (central vein), no signs of necrosis or inflammation was observed; Con-A group, hepatic tissue exhibited notable necrotic and inflammatory changes as specimen displayed marked hepatocytes trabeculation, hydropic degeneration, and apoptotic changes (arrow heads) and several inflammatory cell infiltrations (arrows) and congestion of the central veins (CV); GE 25 + Con-A group, the lesions were remarkably decreased, specimen showed alternating areas of multiacinar necrosis with the same pathological changes with areas of a normal hepatic structure with normal areas; and GE 50 + Con-A group possessed remarkable improvement of the hepatic lesions with still hepatocytes having a vacuolated cytoplasm and a less marked apoptotic nuclei and inflammatory cell infiltration; histopathological lesion scores in liver tissue among different groups. Values are the mean ± SEM (*n* = 8). * *p* ˂ 0.05, ** *p* ˂ 0.01, *** *p* ˂ 0.001 vs. control group; ^#^ *p* ˂ 0.05, ^##^ *p* ˂ 0.01, ^###^ *p* ˂ 0.001 vs. Con A group.

Con-A injection caused marked hepatic injury, as evidenced by considerable raising in all hepato-toxicity serum indices (ALT, AST, ALP, LDH, and γ-GT) and hepatic MPO, comparing to the control group (Figure 2I,II). Furthermore, histopathological examination of the hepatic specimen revealed marked hepatic lesions in the form of extensive inflammatory cell infiltrations, diffuse intense sinusoidal congestion hepatocytes trabeculation, hydropic degeneration, and apoptotic changes (Figure 2III). Interestingly, GE pretreatment prohibited the development of Con-A-caused hepatitis as it lessened all the biochemical indices of hepatic damage, as well as the pathological deterioration of the liver compared to the Con-A group. Moreover, GE suppressed the elevation of MPO level, pointing to the inhibition of neutrophil infiltration into hepatic tissue.

### 3.2. GE Decreased Immuno-Expression of CD4+ and PCNA in Con-A-Challenged Mice

**Figure 3:**
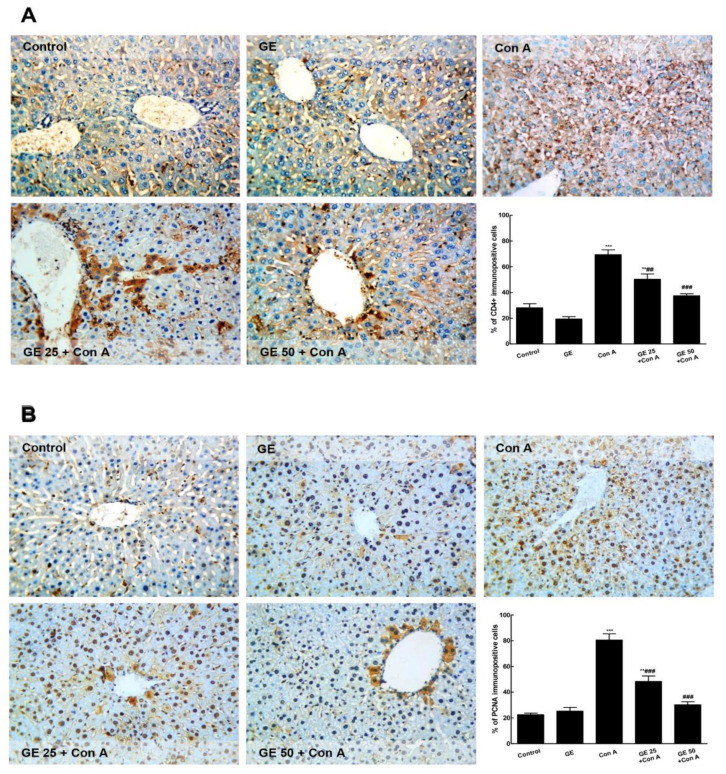
Garcinone E (GE) decreased the immuno-expression of CD4+ and PCNA in concanavalin-A (Con-A)-challenged mice. Immuno-expression of (**A**). CD4+ and (**B**). PCNA. The immuno-staining of CD4+ and PCNA of the control and GE groups was minimal, while that of Con A was high; GE pre-treated groups showed a remarkably lowered level of immunostaining (×200). Values are the mean ± SEM (n = 8). ** *p* ˂ 0.01, *** *p* ˂ 0.001 vs. control group; ^##^ *p* ˂ 0.01, ^###^ *p* ˂ 0.001 vs. Con A group.

As represented in Figure 3, the immuno-expression of CD4+ and PCNA was minimal in the control and GE groups. The Con A challenge induced a significant elevation of the immunostaining of both CD4+ and PCNA. GE pre-treated groups demonstrated a remarkable minimized immunostaining level compared with the Con A group.

### 3.3. GE Enhanced Nrf2 Signaling and Antioxidants to Counteract Con-A-Induced Oxidative Response in Hepatic Tissue

**Figure 4:**
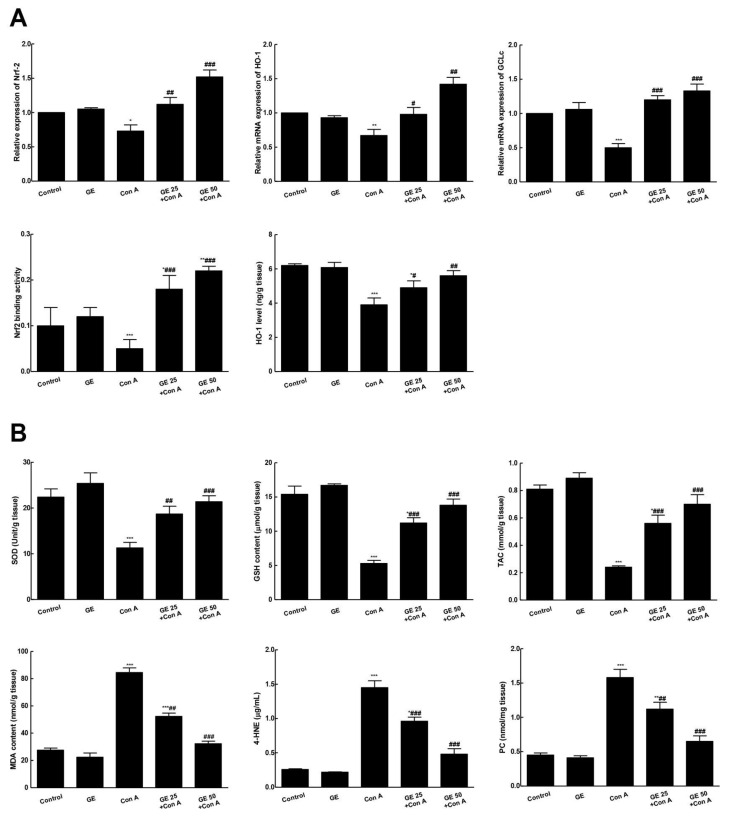
Garcinone E (GE) enhanced Nrf2 signaling and antioxidants to counteract concanavalin A (Con-A)-induced oxidative response in hepatic tissue. (**A**). mRNA expression of Nrf2, HO-1 and GCLc; Nrf2 binding activity; HO-1 level in hepatic tissue; (**B**). Antioxidants and oxidative stress markers: superoxide dismutase (SOD); reduced glutathione (GSH); total antioxidant capacity (TAC); malondialdehyde (MDA); 4-Hydroxynonenal (4-HNE); protein carbonyl (PC). Values are the mean ± SEM (n = 8). * *p* ˂ 0.05, ** *p* ˂ 0.01, *** *p* ˂ 0.001 vs. control group; ^#^ *p* ˂ 0.05, ^##^ *p* ˂ 0.01, ^###^ *p* ˂ 0.001 vs. Con A group.

As shown in Figure 4, Con A depressed Nrf2 mRNA expression and correlated genes (GCLc and HO-1) compared to the control. This was associated with notable repression of Nrf2 binding capacity and a reduced HO-1 level. Modulation of Nrf2 signaling by Con A was simultaneous with antioxidant reduction (SOD, GSH, and TAC) in the hepatic tissue and a raise in the oxidative burden in hepatic tissue as peroxidative marker (MDA, 4-HNE, PC) levels were reinforced, comparing to the control. On the other hand, GE pretreatment notably enhanced Nrf2 signaling, binding activity, and antioxidants and ameliorated the peroxidative markers in the hepatic tissues indicating the suppression of Con-A-associated oxidative stress.

### 3.4. GE Suppressed Con-A-Induced NF-κB Activation and Downstream Inflammation Cascades

**Figure 5:**
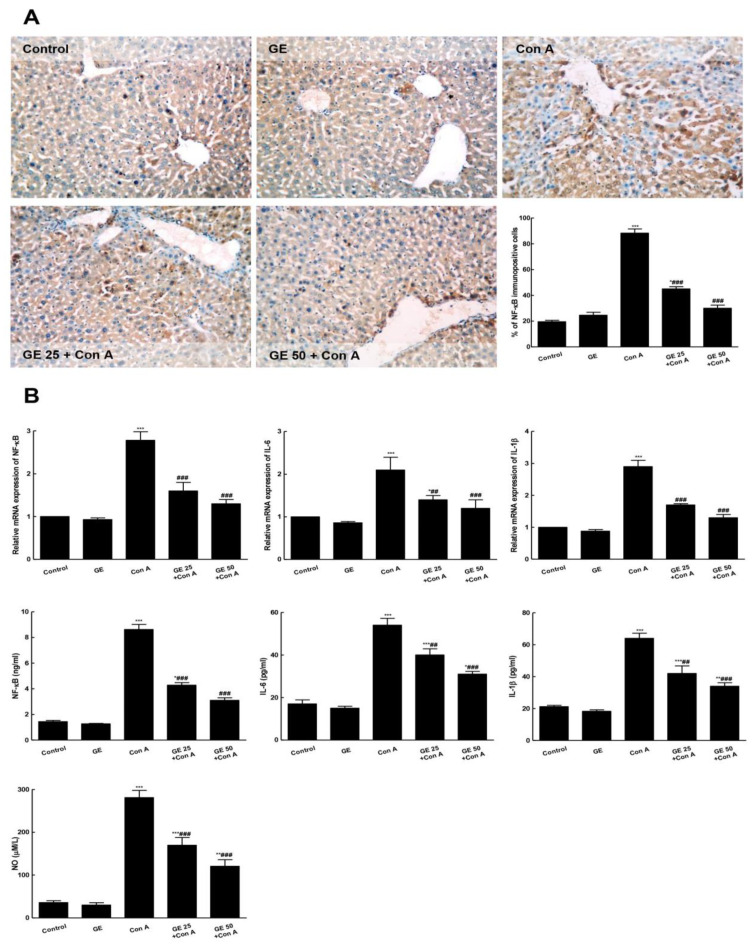
Garcinone E (GE) repressed Con-A-induced NF-κB activation and downstream inflammatory cascade. (**A**). Immuno-stained hepatocytes with NF-ĸB p65 (200×) where control and GE groups have only positive hepatic sinusoidal walls; the Con A group showed extensive immune staining and increased number of immune positive cells, while the treated groups of GE + Con A shown have less marked presentation of immune staining, almost near normal staining; semiquantitative analysis of NF-ĸB IHC staining results expressed as % of NF-ĸB immunopositive cells. (**B**). mRNA expression and level of NF-κB; IL-6; IL-1β in the hepatic tissue. Values are the mean ± SEM (n = 8). * *p* ˂ 0.05, ** *p* ˂ 0.01, *** *p* ˂ 0.001 vs. control group; ^##^ *p* ˂ 0.01, ^###^ *p* ˂ 0.001 vs. Con A group.

Con-A injection resulted in an increase in the NF-κB immuno-expression and level compared to control mice that was concurrent with an elevated level of IL-6 and -1β and NO, compared to the control (Figure 5). Contrarily, GE interrupted NF-κB activation as the immuno-stained hepatocytes and its level were attenuated. Additionally, the levels of ILs (-6 and 1β) and NO were significantly reduced compared to the Con-A group.

### 3.5. GE Inhibited TNF-α/JNK Signaling and Ameliorated Con-A-Induced Apoptosis 

**Figure 6:**
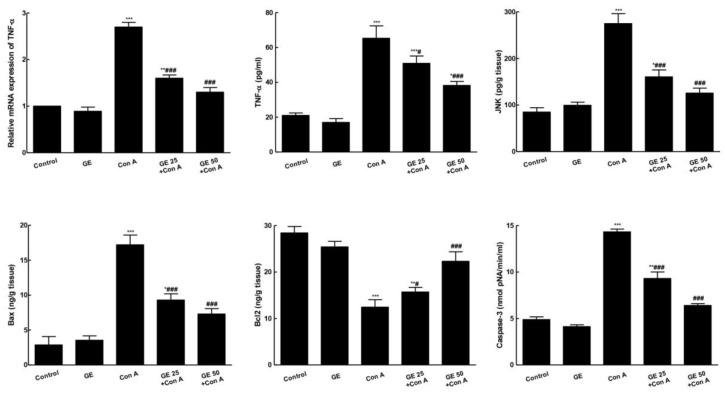
Garcinone E (GE) inhibited TNF-α/JNK signaling and ameliorated concanavalin A (Con-A)-induced apoptosis. Values are the mean ± SEM (n = 8). * *p* ˂ 0.05, ** *p* ˂ 0.01, *** *p* ˂ 0.001 vs. control group; ^#^ *p* ˂ 0.05, ^###^ *p* ˂ 0.001 vs. Con A group.

Con A noticeably enhanced the mRNA and level of TNF-α, as well as the level of JNK. This was accompanied by an augmented level of apoptotic markers (Bax, caspase-3) in the Con-A group (Figure 6). Simultaneously, Con-A significantly reduced the anti-apoptotic protein (Bcl2) level, compared to the control. In contrast, GE pretreating hindered TNF-α/JNK signaling and Con-A-induced apoptotic changes as it lowered Bax and caspase-3 hepatic levels simultaneously, with notable elevation in the Bcl2 hepatic level.

## 4. Discussion

Garcinone E (GE), a xanthone derivative reported from *G. mangostana,* possesses cytotoxic and advanced glycation end-products and aromatase-inhibitory activities [38,39,40]. The present study is the first to investigate the GE hepatoprotective capacity on Con-A-induced AIH. The results reveal that the potent anti-inflammatory, antioxidant, and anti-apoptotic activities of GE were protected when compared to Con-A-caused hepatitis. These activities were correlated to GE’s ability to enhance HO-1/Nrf2/antioxidant signaling and to inhibit the TNF-α/NF-κB/JNK axes.

Con-A-induced hepatitis is a well-established model that resembles human acute viral hepatitis and AIH regarding to its pathogenesis [41,42]. Single Con-A injection can speedily produce grave immune-mediated inflammatory liver damage within 8–12 h [7,36]. Con A has a high tendency towards mannose-wealth glycoproteins on liver sinusoidal endothelial cells, leading to T cell activation, particularly the CD4+ T cells in the liver tissue. The activation of cells stimulates the secretion of many cytokines (e.g., TNF-α, ILs, and IFN-γ) involved in mediating inflammation and in cell–cell communication [10,43]. The occurrence of Con-A-induced hepatitis was confirmed by our biochemical, histopathological, and IHC results. Serum ALT, AST, ALP, LDH, and γ-GT are cytosolic enzymes that are freed into the blood after hepatocyte death, and hence they may be considered biochemical indices of hepatotoxicity. Our results indicated a significant increase in these markers in the serum of the Con-A-intoxicated group, indicating hepatic injury. Histopathological examination confirmed extensive deteriorative hepatitis in the Con A group. Additionally, the MPO level was remarkably raised in the hepatic tissue confirming the infiltration of neutrophils. Besides, Con-A-induced recruitment of CD4+ T cells into the liver was obvious in IHC results which interplay with other inflammatory cells to mediate the cellular inflammatory response. Cellular injury stimulates nuclear repair to enhance cellular recovery. Hence, an elevated level of nuclear PCNA indicates the occurrence of hepatocellular injury. Our IHC results revealed the number of PCNA-positive hepatocytes in the Con-A group and confirmed the incidence of hepatitis in Con-A-challenged mice. All these injurious changes are in assent with many previous studies [44,45,46]. Contrarily, the GE-pretreated animals showed noticeable amendment in all histopathological and biochemical indices of hepatic hurt, revealing the potent hepato-protection capacity of GE. Additionally, GE counteracted the infiltration of inflammatory cells and CD4+ T cells recruitment into the liver which suggested the anti-inflammatory activity of GE. 

The etiology of Con-A-induced hepatitis is multifactorial and includes many molecular pathogenic pathways. One of these is the suppression of the Nrf2/GCLc/HO-1 signaling resulting in the downregulation of the antioxidant enzymes and an increase in the oxidative burden [36]. Furthermore, the increased release of reactive-oxygen-species (ROS) for 12 h next to Con-A injection was found to augment lipid peroxidative injury and to greatly repress the liver’s antioxidant capacity [46,47]. This state of oxidative damage strengthens inflammatory cytokine expression, likely through activating such intracellular diverse inflammation signaling pathways as NF-ĸB, resulting in aggravation of injury and inflammation [5,7,9,48]. The obtained results are in compliance with the former ones as they further assured the down-regulation of Nrf2 and its downstream genes (HO-1/GCLc) following Con A injection. That was accompanied by a remarkable depression of antioxidants (e.g., GSH, SOD, and TAC) in the Con A group’s liver. Additionally, the liver experienced peroxidative damage as there was an elevation in the lipid-peroxidative end products (4-HNE, MDA, and PC) in the Con A group’s liver. Notably, GE pretreatment enhanced Nrf2 signaling and so increased the levels of hepatic antioxidants which resulted in a diminution of PC, MDA, and 4-HNE. These data provided evidence that Nrf2 activation may be responsible in part for GE hepato-protective activity.

Inflammation represents the prime etiology of Con-A-caused liver damage, while NF-ĸB pathway activation is the main mediator of the cellular reaction to Con A as it encourages inflammation cytokines’ release and expression [2,6,49]. Moreover, NF-ĸB signaling leads to NOx overproduction due to enhanced expression of iNOS, which overstresses Con-A-induced hepatitis [5]. In line with the abovementioned investigations, our results assured NF-ĸB activation and subsequent IL1β, IL6, and NO release following the Con A challenge. Contrarily, GE pretreatment notably impeded NF-ĸB activation and lessened inflammatory cytokine levels. 

TNF-α is a central inflammatory mediator that controls Con-A-deleterious necrotic and apoptotic damage. Following Con-A injection, Kupffer cells and T cells are greatly activated and infiltered in hepatic tissue causing immunological liver damage [7,41,42]. These cells over-secrete ROS, ILs, and TNF-α which binds to TNF- receptor-1 existing in the hepatocytes, leading to the activation of various signal transduction-linked proteins such as JNK that modulates the survival or death of hepatocytes during the inflammatory reaction [50]. An earlier study has shown that JNK-deficient mice are resistant to Con-A-caused hepatitis [51]. Additionally, TNF-α induces the activation and release of other inflammatory mediators, leading to the augmentation of the inflammatory response and recruitment of other immune cells [52]. Another apoptotic pathway that happens alongside the TNF-α/JNK axis is the TNF-α-dependent activation of caspase-8 and -3 which results in hepatocyte apoptosis [16]. Furthermore, Con A injection induces the elevated expression of proapoptotic proteins: Bax and caspase-8. More recently, anti-apoptotic protein (Bcl2 and Bcl-xl) prohibition and pro-apoptotic protein (e.g., Bad and Bax) elevation have been noticed following Con A injection [11,53]. The imbalance between anti- and pro-apoptotic proteins in Con-A-treated animals was attributed to modulation in NF-ĸB and Nrf2 signaling pathways leading to hepatocyte apoptosis [8,9]. Our findings asserted the elevated TNF-α and JNK expression and levels that were correlated with increased apoptotic markers (caspase-3 and Bax) and decreased anti-apoptotic marker Bcl2 in the Con-A group. These changes were significantly reversed in GE-pretreated animals. Hence, it may be suggested that GE-protective effects are mediated through the inhibition of TNF-α/JNK/apoptosis signaling.

## 5. Conclusions

**Figure 7:**
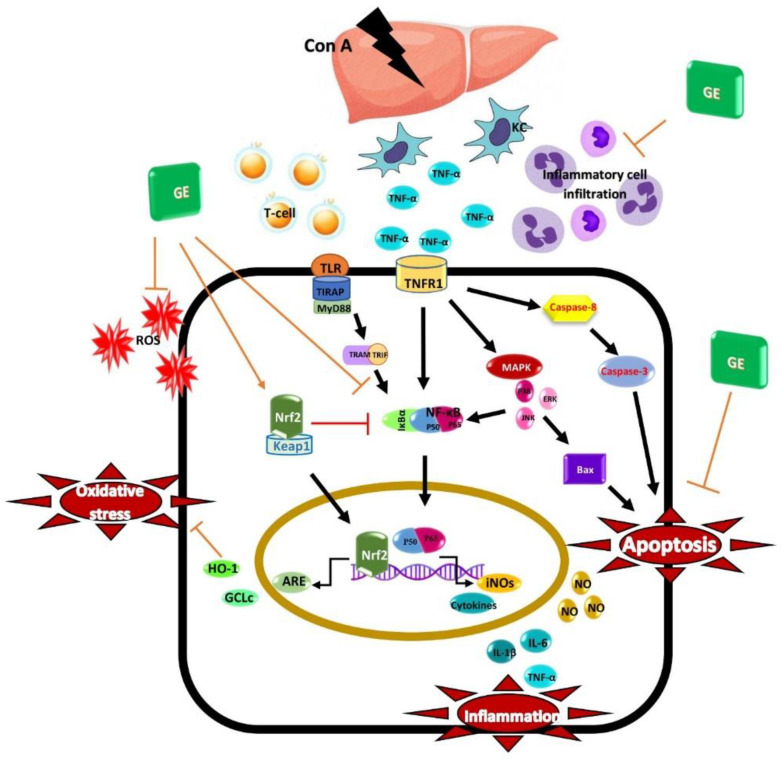
Possible molecular mechanisms of the hepatoprotective activity of garcinone E (GE) against concanavalin A (Con-A)-induced AIH.

Our study demonstrates the powerful GE hepato-protection capacity on Con-A-induced AIH that may be connected to its anti-inflammation, anti-oxidant, and anti-apoptotic abilities. GE modulated HO-1/Nrf2 signaling and suppressed the activation of NF-κB/inflammatory cascade alongside the TNF-α/JNK/apoptosis axis (Figure 7). These data suggest GE’s potential usage against AIH as a novel hepato-protective candidate.

## Figures and Tables

**Table 1:** The primers used in RT-PCR.

Gene (Mouse)	Accession	Sequence (5′-3′)	PCR Product Size (bp)
*Nrf2*	NM_010902	**F**: TGAAGCTCAGCTCGCATTGA **R**: TGCTCCAGCTCGACAATGTT	108
*HO-1*	NM_010442	**F**: GAAATCATCCCTTGCACGCC	122
		**R**: CCTGAGAGGTCACCCAGGTA	
*GCLc*	NM_010295	**F**: GCTTTGGGTCGCAAGTAGGA	181
		**R**: GCGTCCCGTCCGTTCC	
*NF-ĸB*	NM_008689	**F**: CCACTGTCAACAGATGGCCC	158
		**R**: TTGCAAATTTTGACCTGTGGGT	
*IL-6*	NM_031168	**F**: CCCCAATTTCCAATGCTCTCC	141
		**R**: CGCACTAGGTTTGCCGAGTA	
*IL-1β*	NM_008361	**F**: TGCCACCTTTTGACAGTGATG	138
		**R**: TGATGTGCTGCTGCGAGATT	
*TNF-α*	AY423855	**F**: TCCCAAATGGCCTCCCTCTC	98
		**R**: TACGACGTGGGCTACAGGCT	
*β-actin*	NM_007393	**F**: CTGAGCTGCGTTTTACACCC	200
		**R**: CGCCTTCACCGTTCCAGTTT	

## Data Availability

Not applicable.
